# The Safety of Laparoscopic Cholecystectomy in the Day Surgery Unit Comparing with That in the Inpatient Unit: A Systematic Review and Meta-Analysis

**DOI:** 10.1155/2020/1924134

**Published:** 2020-04-28

**Authors:** Wei Xiong, Ming Li, Ming Wang, Shu Zhang, Qin Yang

**Affiliations:** ^1^Department of Hepatobiliary Surgery, Sichuan Provincial People's Hospital, Chengdu, 610000 Sichuan Province, China; ^2^Department of General Surgery, Chengdu Third People's Hospital & the Affiliated Hospital of Southwest Jiaotong University & the Second Medical School of Medical School of Chengdu Affiliated to Chongqing Medical University, Chengdu, Sichuan 610031, China

## Abstract

We aimed to perform a systematic review and meta-analysis on the safety of laparoscopic cholecystectomy performed in the day surgery unit versus those performed in the inpatient unit. Several databases including Ovid Embase, Medline Ovid, Cochrane Central, Web of Science, and Google Scholar were searched from inception through February 2019. Our results revealed that laparoscopic cholecystectomy can be conducted safely and effectively in day surgery units, helping bed shortage.

## 1. Introduction

Laparoscopic cholecystectomy (LC) is considered the “gold standard” for the surgical treatment of gallstone disease because it results in less postoperative pain, better cosmesis, and shorter hospital stays and recover faster than open cholecystectomy [1–5]. Currently, approximately 750,000 laparoscopic cholecystectomies are performed annually in the United States, which accounts for roughly 90 percent of all cholecystectomies [6, 7]. Routine LC requires patients to admit at least one night in the inpatient units. With the development of day surgery, patients with good home support can leave the hospital within six hours after surgery [8, 9].

Day surgery LC is known for many benefits, including overcoming inpatient bed shortage and cost effective, compared to routine LC [10, 11]. However, the growth of day surgery LC is till slow and even has not developed in most developing countries like China mainland [12, 13]. Concerns about safety, not the lack of adequate techniques and facilities, have curbed the wide-scale development of LC as day surgery [14, 15]. Thus, we aim to assess the safety of LC in day surgery units through an updated systematic review and meta-analysis, which will raise an important reference value to promote the establishment and development of day surgery LC in developing countries.

## 2. Methods

### 2.1. Literature Search

The search strategy and subsequent literature search were developed and performed with the assistance of an experienced medical reference librarian (W.M.B) following five “PICOS” components (supplementary S1). The search strategies were developed in Ovid Embase and translated to match the subject headings and keywords for Medline, Ovid, Cochrane Central, Web of Science, and Google Scholar, from inception through February 2019. The following items were used: day surgery, day-case surgery, day stay, hospitalization, outpatient surgery, ambulatory surgery, and laparoscopic cholecystectomy. The details of the search strategy are provided in Supplementary S2. Electronic searches were supplemented by manual searches for references to the included studies and review articles. All results were downloaded from a bibliographic database manager, EndNote 9.0 (Thomson ISI ResearchSoft, Philadelphia, Pennsylvania, USA).

### 2.2. Selection

A single reviewer (W.X) screened the titles and abstracts. Full articles were assessed by two pairs of independent reviewers (W.X and M.W), and discrepancies were resolved through adjudication.

Inclusion criteria were (1) studies compared day surgery LC with inpatient with randomization or not and case series about day surgery LC with more than 10 patients; (2) studies included patients who consented to participate day surgery LC before operation; (3) day surgery was defined as patients underwent operation and discharged within the same day, and overnight stay was defined as patients stayed more than one night after operation; (4) adult patients aged younger than 75 years and with BMI less than 35; (5) patients with no significant comorbidities before LC; and (6) patients did not have history of open abdominal surgeries.

Exclusion criteria were (1) studies that did not meet the inclusion criteria for day surgery or no definite criteria for day surgery, (2) patients that had previous abdominal surgery, (3) no definition or differentiation between inpatient and outpatient surgery, (4) with no relevant data or insufficient data, (5) sample size of fewer than 10 patients, (6) animal studies or non-English-language articles, (7) laparoscopic cholecystectomy for pregnant or diabetic patients, and (8) letters, comments, conference abstracts, and reviews.

### 2.3. Quality Assessment

This study was conducted following the preferred reporting items for systematic review and meta-analysis (PRISMA). The Cochrane Collaboration tool was used to assess the quality of the RCTs by two reviewers independently. All different opinions about quality assessment were discussed with a third reviewer (Q.Y) to reach an agreement on consensus.

### 2.4. Data Extraction

Study characteristics were extracted from two reviewers (M.W and W.X) with structured data extraction forms, including study design, country, year of publication, sample size, diseases at LC, patient demographics, American Society of Anesthesiologists (ASA) scores, trial duration, interview time, postoperative complications, postoperative nausea and vomiting (PONV), Visual Analogue Score (VAS), discharge time, time to normal activity, operation time, readmission, patients' satisfaction, and total cost. Outcome measures including percentages, mean, or median values with standard deviations or ranges were recorded.

Any disagreement between reviewers was to be discussed with a third reviewer (Q.Y) to reach an agreement. The corresponding author of the identified paper was contacted to request incomplete or unpublished data.

### 2.5. Statistical Analysis

Heterogeneity across the studies was assessed using the *Q* statistic test and *I*^2^ statistic. The presence of heterogeneity was considered significant if the *p* value of the *Q* test was less than 0.01 or the *I*^2^ value was more than 50%. If the interstudy heterogeneity was significant, a DerSimonian-Laird random-effects model was used, or a fixed-effects model was conducted. Pooled Risk Ratio (RR) or Odds Ratio (OR) with 95% confidence interval (CI) was estimated for dichotomous data, and Mean Difference (MD) with 95% CI was estimated for continuous data. All statistical analyses were performed using RevMan software (version 5.3, Cochrane Collaboration). Statistical significance was assessed at the *α* = 0.05 level.

## 3. Results

### 3.1. Search Results

**Figure 1:**
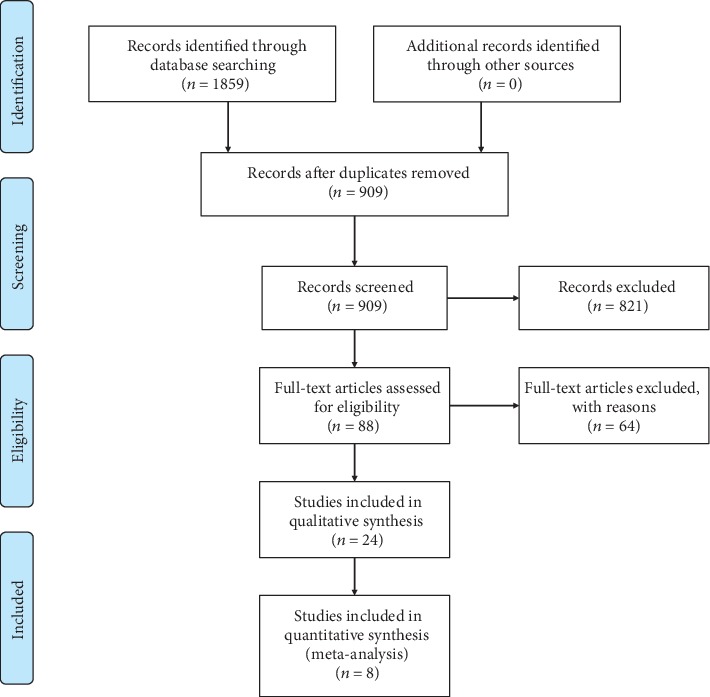
Preferred reporting items for systematic reviews and meta-analysis (PRISMA) flow chart for this study.

In total, 1859 unique articles were included after the search of several databases. Eighty-eight articles were identified for full-text review after the title and abstract screening, and 64 of them were excluded with reasons. The remaining 24 articles included 8 RCTs for quantitative synthesis and the other 16 case series or controlled studies only for qualitative analysis (Figure 1).

### 3.2. Baseline Characteristics

The eligible 24 studies including 8 RCTs and 14 case series and 2 retrospective controlled studies were published from 1998 to 2018 [16–39]. These studies reported the day surgery LC performed on cholelithiasis or gallbladder polyps with the trial duration ranged from 6 months to 72 months. Only two studies [24, 25] reported more male patients underwent day surgery LC (90% and 52% males, respectively). The female patients accounted for 52% to 88.5% in the other 22 studies (Table 1).

There were 10 studies [32–39] that compared the LC in the day surgery unit versus inpatient unit, and 2 of them [18, 26] were retrospective controlled studies which were excluded from quantitative synthesis. Of the remaining 8 RCTs, there were 301 patients that underwent LC in day surgery units and 308 patients underwent LC in the inpatient units. All patients were given prophylactic analgesia, except that data was not available in the two studies. All the patients in the day surgery group were discharged 4-8 hours after surgery if they meet the discharge criteria, and the patients in the inpatient group were scheduled to discharge the following day after surgery (Table 2).

### 3.3. Outcomes

#### 3.3.1. Postoperative Complications

**Figure 2:**
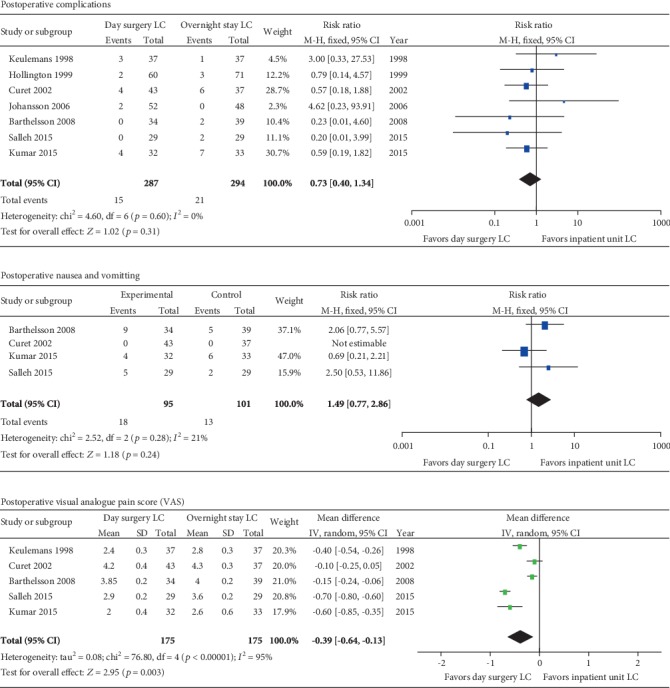
Meta-analysis forest plot concerning postoperative complications. VAS: significant difference in favor of day surgery unit versus inpatient unit. There was no significant difference of postoperative complications and PONV between day surgery and inpatient groups.

Postoperative complications were reported in seven trials [32, 33, 35–39] with 287 patients in the day surgery group and 294 patients in the inpatient group. In total, 15 out of 287 (5.2%) participants had postoperative complications in the day surgery group compared with 21 out of 294 (7.1%) in the inpatient group. The pooled RR was 0.73 with 95% CI 0.4-1.34, and no significant difference was observed (*p* = 0.3) (Figure 2).

#### 3.3.2. Postoperative Nausea and Vomiting (PONV) and Visual Analogue Score (VAS)

There were three studies [37–39] that reported the PONV with 196 participants in total. One study reported a higher PONV rate in the inpatient group (18.2% vs. 12.5%) than that in the day surgery group. The other two studies reported higher PONV rates in the day surgery group (26.5% vs. 12.8%; 17.2% vs. 6.9%) than those in the inpatient group. The heterogeneity was not significant (*p* = 0.28; *I*^2^ = 21%), and the pooled RR was 1.49 with no significant difference between two groups (*p* = 0.24; 95% CI 0.77-2.86) (Figure 2).

Five trials [32, 35, 37–39] assessed the VAS of patients after operation, including 175 patients in each group. The heterogeneity was significant (*p* < 0.001; *I*^2^ = 95%), but the exclusion of any one of the five studies did not change the results. So, the random model was used to estimate the pooled effect (MD = −0.39; 95% CI -0.64, 0.13), and the VAS in the day surgery group was significantly lower than that in the inpatient group (*p* = 0.003) (Figure 2).

#### 3.3.3. Prolongation of Hospital Stay

**Figure 3:**
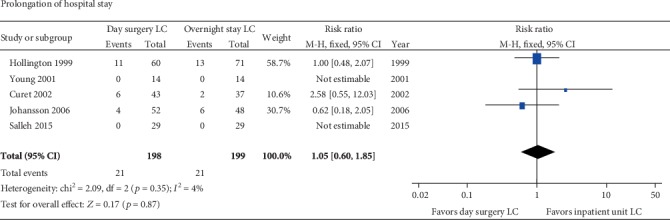
Meta-analysis forest plot concerning the prolongation of hospital stay. No significant difference was observed between two groups (day surgery group versus inpatient group).

The rate of successful discharge of patients after day surgery LC ranged from 82% to 100% in all the included studies (Table 1). Five RCTs [33–36, 39] including 397 patients reported the prolongation of hospital stay. Two of them reported nil prolongation in each group, and the other three studies had 5.4% to 18.3% of patients that prolonged the hospital stay. There was no heterogeneity (*p* = 0.35; *I*^2^ = 4%) between these five trials, and the pooled RR was 1.05 (95% CI 0.6-1.85) with no significant difference (*p* = 0.87) (Figure 3).

#### 3.3.4. Return to Normal Activity

**Figure 4:**
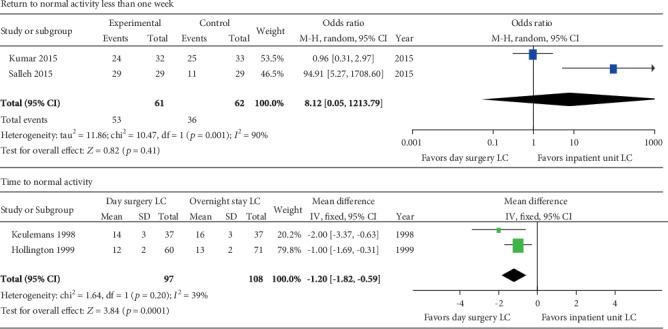
Meta-analysis forest plot concerning time to return to normal activity. Time to normal activity: significant difference in favor of day surgery versus inpatient unit.

There were four studies reported that patients returned to normal activity or those returned to work after surgery. Two of them [32, 33] showed the time of patients return to normal activity after surgery, and the other two [38, 39] reported the number of patients who returned to normal activity less than one week. So, we analyzed them separately and estimated the OR for dichotomous data and MD for continuous data. The two studies with patient number had significant heterogeneity (*p* = 0.001). The similar percentage of patients who returned to normal activity less than one week in the two groups (day surgery 75% versus overnight stay 75.8%) was reported in one study [38] while a much higher percentage of that in the day surgery group was observed in the other study (day surgery 100% versus inpatient 37.9%) [39]. The pooled OR (OR = 8.12; 95% CI 0.05-1213.9) was calculated from the randomized model, and the overall effect was not significant (*p* = 0.41) (Figure 4).

The other two studies [32, 33] showed that patients took a shorter time to return to normal activity for patients in the day surgery group than those in inpatient group (MD -1.2; 95% CI -1.82, -0.59; *p* = 0.0001), and there was no significant heterogeneity (*p* = 0.2; *I*^2^ = 39%) between these two studies (Figure 4).

#### 3.3.5. Patients' Satisfaction

**Figure 5:**
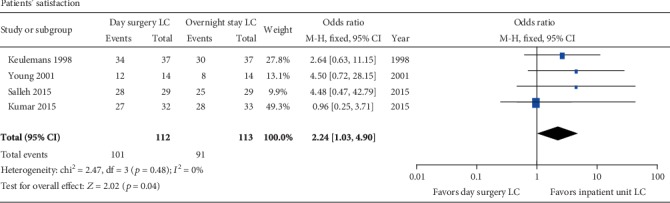
Meta-analysis forest plot concerning patients' satisfaction. Significant difference in favor of inpatient unit versus day surgery unit after LC.

The patients' satisfaction of day surgery LC varied from 78% to 97% for all the included studies (Table 1). Five trials [32–35, 38, 39] compared the patients' satisfaction in the day surgery group with that in the inpatient group. One of them [35] showed satisfaction scores with mean 3.4 in the day surgery group and 3.1 in the inpatient group. The other four studies reported the patients' number of satisfaction with surgery. These four trials had no significant heterogeneity (*p* = 0.48; *I*^2^ = 0%). The patients' satisfaction rate in the day surgery group was significantly higher than that in the inpatient group (RR = 2.24; 95% CI 1.03-4.9; *p* = 0.04) (Figure 5).

#### 3.3.6. Readmission and Cost

**Figure 6:**
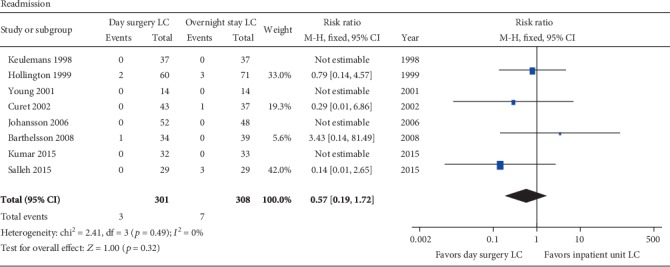
Meta-analysis forest plot concerning readmission. No significant difference between two groups.

All the included RCTs reported patients' readmission, and 4 of them [32, 34, 36, 38] reported 0% readmission in both groups. The other 4 trials [33, 35, 37, 39] reported 0% to 3.3% readmission rates in the day surgery group and 0% to 10.3% readmission rates in the inpatient group. There was no significant heterogeneity among these studies (*p* = 0.49; *I*^2^ = 0%). The pooled effect was not significantly different between the two groups (RR = 0.57; 95% CI 0.19, 1.72; *p* = 0.32) (Figure 6).

**Figure 7:**
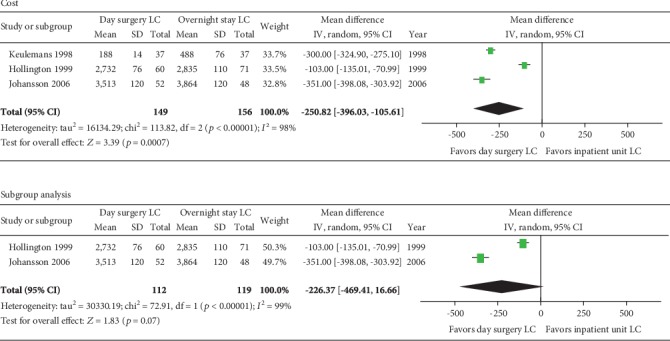
Meta-analysis forest plot concerning cost. Significant difference in favor of day surgery versus inpatient unit.

Three RCTs [32, 33, 36] reported the cost of LC as day surgery and overnight stay procedure. All the three trials reported less cost by day surgery LC than by inpatient LC, and the pooled effect was significantly different (MD -250.8; 95% CI -396, -105.6; *p* = 0.0007). However, the heterogeneity among them was significant (*p* < 0.001) and one study [32] affected the stability of the overall effect obviously. So, the subgroup analysis without this study was performed, and the reestimated result showed that there was no significant difference between day surgery and inpatient LC (*p* = 0.07) (Figure 7).

### 3.4. Quality Assessment

The quality of the 8 RCTs was assessed with the Cochrane Collaboration tool, and the risk of bias was shown as Figure S1 in the supplementary. Six of the 8 RCTs reported the randomization sequence, and 8 of them reported the allocation concealment. None of them reported blinding.

## 4. Discussion

This study was conducted to assess the safety and benefits of LC as a day surgery procedure compared to an inpatient procedure. Our results showed that LC can be performed safely and cost effectively in day surgery units for selective patients. Similar postoperative complications, PONV, and prolongation of hospital stay and readmission rates of patients after LC were observed in the day surgery group and the inpatient group. Significant smaller VAS and shorter time to return to normal activity were observed in the day surgery group than those in the inpatient group, which could increase the patients' satisfaction. Besides, the inclusion criteria for selective patients, easy reach to the hospital, and the schedule of operation which should be in the morning are also required for day surgery LC.

The LC can be performed safely in both inpatient units and day surgery units.

There were rare severe complications after LC in either day surgery units or inpatient units. The most common postoperative complications included fever, wound infection, diarrhea, PONV, and pain. The postoperative complication rates were similar in the day surgery group (5.2%) with that in the inpatient group (7.1%). PONV and postoperative pain are common in patients after LC and directly influence the hospital stay, readmission, and postoperative satisfaction. Six of the 8 RCTs [32, 35–39] performed prophylactic analgesia to relieve PONV and postoperative pain in this study, and 12.5%-26.5% of patients experienced PONV in the day surgery group compared to 6.9%-18.2% of patients in the inpatient group. But there was no significant difference between the two groups in PONV (RR = 1.49; *p* = 0.24; 95% CI 0.77-2.86), which was consistent with the previous study [40, 41]. The mean VAS after LC was lower in the day surgery group than that in the inpatient group. Five RCTs in this study reported VAS within 24 h after LC, and the MD was estimated as -0.39 (95% CI -0.64, 0.13; *p* = 0.003). In other words, patients in the day surgery group had less postoperative pain than those in the inpatient group. The patients in the day surgery group might feel more comfortable at home the night after surgery so that they got lower VAS than those staying in the hospital in the inpatient group. Readmission rate is another critical factor to assess the safety of the surgical procedure. In this study, 8 RCTs [32–39] reported the readmission rate after discharging ranged from 0% to 10.3% in total. The main reasons for readmission are still PONV, fever, pain, and wound infection. Therefore, we revealed that patients that underwent LC in the day surgery units had lower VAS than those in the inpatient units.

The LC can be performed as a day surgery procedure effectively. In total, 82%-100% of the patients after LC discharged the same day successfully in the 24 studies included in this study. The main reasons for prolonging the hospital stay include postoperative pain, PONV, and conversion to open surgery [30, 31, 42]. In addition, some patients that lived far from the hospital would choose to prolong the hospital stay, and similar rates were observed in both day surgery and inpatient groups. The rates of prolongation of hospital stay are both about 0% to 18% in the two groups, and there was no significant difference between them (RR = 1.05; 95% CI 0.6-1.85; *p* = 0.87). Most patients can return to normal activities or work within one week either in the day surgery group or in the inpatient group. One study [38] reported that 75% of patients returned to work after LC in both groups. However, the other study [39] reported 100% of patients in the day surgery group versus 28% of patients in the inpatient group who returned to work less than one week. Another two studies [32, 33] reported the time to normal activity, and we estimated the pooled MD. The result revealed that patients after LC in the day surgery units took less time to normal activity than those in the inpatient units (*p* = 0.0001). Given the above, patients after LC in the day surgery units need less recovery time than those in the inpatient units; thus, it is effective to perform LC as a day surgery procedure.

Day surgery LC increased patients' satisfaction but did not reduce cost significantly compared to the overnight stay procedure. The satisfaction rate of patients after day surgery LC ranged from 78% to 97%, and it was not significantly different to those in the inpatient units, which was consistent with the previous study. There were 6 studies [23, 24, 27, 36, 38, 39] that reported less cost of LC in day surgery units than that in the inpatient units. However, we quantitatively analyzed the two RCTs and the results showed no significant difference between the two groups (*p* = 0.07). However, in our opinion, the day surgery saved the bed cost to reduce the total cost and more studies would be required to document this conclusion.

In conclusion, our results revealed that patients that underwent LC in the day surgery units had similar postoperative complications, readmission rates, and prolongation of hospital stay comparing with those in the inpatient units, and these results were consistent with previous studies [40, 42, 43]. Furthermore, our study firstly revealed that patients after LC had lower VAS in the day surgery group than that in the inpatient group. Furthermore, we firstly estimated the cost of these two procedures, and the result revealed less cost in day surgery units than that in the inpatients units with no significant difference. Although we only synthesized RCTs with no significantly different results, three other non-RCT studies [23, 24, 27] had reported less cost for day surgery LC. Thus, we believe that LC performed in day surgery units is cost effective than routine LC procedures.

## Figures and Tables

**Table 1:** Characteristics of the included studies.

First author	Year	Study type	Country	Diseases	Duration (mo.)	DSLC no.	Age (year)	Female no. (%)	Operation time (min)	Discharge, no. (%)	Satisfaction (%)
*Studies only for qualitative analysis*											
Rabi [31]	2018	Case series	Pakistan	Cholelithiasis	12	113	37.9 ± 8.5	100, 88.5%	44.5 ± 12.8	99, 87.6%	NA
Tiryaki [30]	2016	Case series	Turkey	Symptomatic chronic cholelithiasis	48	60	40.6 ± 8.1	45, 75%	NA	55, 91.7%	89.6%
Al-Qahtani [29]	2015	Case series	Saudi Arabia	Symptomatic cholelithiasis	48	487	41.9 ± 8	426, 87.5%	NA	465, 95.5%	96.90%
AI-Omani [28]	2015	Case series	Saudi Arabia	Symptomatic cholelithiasis	60	1140	34.2 ± 8	1004, 88%	40.6 ± 12	1094, 96%	NA
Gelmini [27]	2013	Case series	Italy	Gallstone	36	43	49 ± 11.7	28, 65	48.9 ± 14.9	37, 86%	95.30%
Sato [26]	2012	Controlled study	Japan	Symptomatic gallstone	21	50	53.6 ± 14.5	26, 52%	108.4 ± 41.7	41, 82%	NA
Zarour [25]	2009	Case series	Qatar	Symptomatic gallbladder diseases	24	56	NA	27, 48%	NA	48, 85.7%	78.60%
Ali [24]	2009	Case series	Pakistan	Symptomatic cholelithiasis	18	50	43 ± 13.3	5, 10%	NA	46, 92%	NA
Victorzon [23]	2007	Case series	Finland	Not mentioned	72	567	48 ± 9.5	419, 74%	56 ± 18	356, 62.8%	NA
Chauhan [22]	2006	Case series	India	Symptomatic gallstone	12	287	37 ± 7.7	NA	NA	270, 94.1%	NA
Bueno [21]	2006	Case series	Spain	Symptomatic cholelithiasis	62	448	53.1 ± 14.5	338, 75.4%	45 ± 22.4	397, 88.6%	NA
Chok [20]	2005	Case series	China	Symptomatic gallbladder diseases	28	135	47.5	97, 72%	69^#^	125, 92.6%	NA
Barut [19]	2005	Case series	Turkey	Symptomatic cholelithiasis	60	70	37.8 ± 9.9	55, 78.6%	36.6 ± 6.3	70, 100%	NA
Sharma [18]	2004	Controlled study	UK	Cholelithiasis	6	42	47.8 ± 9.6	32, 76.2%	35 ± 12	41, 97.6%	88.10%
Fassiadis [17]	2004	Case series	UK	Symptomatic cholelithiasis	30	100	44 ± 10.2	91, 91%	38 ± 9.2	99, 99%	92.60%
Siu [16]	2001	Case series	China	Symptomatic gallbladder diseases	26	60	40.5 ± 10.5	39, 65%	45.8 ± 21.6	54, 90%	78%
*Studies for quantitative synthesis*											
Salleh [39]	2015	RCT	Malaysia	Symptomatic gallstones	12	29	49.8 ± 13	21, 67.7%	NA	29, 100%	96.6%
Kumar [38]	2015	RCT	India	Symptomatic gallstones	NA	32	35.9 ± 12.4	26, 81%	NA	31, 96.9%	84.40%
Barthelsson [37]	2008	RCT	Sweden	Cholelithiasis	40	34	44.2 ± 10.6	NA	NA	34, 100%	NA
Johansson [36]	2006	RCT	Sweden	Gallstone	12	52	NA	NA	NA	48, 92.3%	NA
Curet [35]	2002	RCT	USA	Symptomatic cholelithiasis	19	43	34.7 ± 11	33, 76.7%	65 ± 15	37, 86%	NA^@^
Young [34]	2001	RCT	Australia	Not mentioned	10	14	38.3 ± 6.4	NA	NA	14, 100%	85.7%
Hollington [33]	1999	RCT	Australia	Not mentioned	20	60	49 ± 14.3	NA	NA	49, 81.7%	NA
Keulemans [32]	1998	RCT	Netherlands	Symptomatic cholelithiasis	22	37	39.4 ± 9.7	28, 70%	76 ± 5	34, 91.9%	92%

The age and operative time were shown in mean ± standard deviation; ^@^it was said more than 90% in the original article; ^#^median operative time; mo.: month; no.: number; DSLC: day surgery laparoscopic cholecystectomy; duration indicates the trial period; discharge indicates patients discharged on the same day within the operation.

**Table 2:** Characteristics of the included studies in the meta-analysis.

Study	Group	No. initially randomized	No. accomplished the trial	Age	Prolong stay	Prophylactic analgesia	Cholangiogram	Discharge
Salleh et al. [39]								
	DS	31	29	49.8 (21-75)	0%	Yes	Selectively	After 6 hours of postsurgical observation
	OS	31	29	49.8 (21-75)	0%			The following day after surgery
Kumar et al.[38]								
	DS	32	32	35.94 ± 12.4^#^	3.1%	Yes	NA	After 6-8 hours of postsurgical observation
	OS	33	33	42.72 ± 11.9^#^	NA			Stay more than one night
Barthelsson et al. [37]								
	DS	50	34	44 (22-68)	0%	Yes	Routinely	After 5-6 hours of postsurgical observation
	OS	50	39	45 (22-68)	NA			The next morning
Johansson et al. [36]								
	DS	54	52	(18-70)	7.7%	Yes	Routinely	After 4-8 hours of postsurgical observation
	OS	53	48	(18-70)	12.5%			The following day after surgery
Curet et al. [35]								
	DS	43	43	33 (18-68)^∗^	14%	Yes	Selectively	After 4 hours of postsurgical observation
	OS	37	37	43 (19-66)^∗^	5.4%			The following day after surgery
Young and O'Connell [34]								
	DS	14	14	39 (26-48)	0%	NA	None	Within 8 hours after surgery
	OS	14	14	40 (21-50)	0%			23 h postsurgery
Hollington et al. [33]								
	DS	74	60	45 (17-83)	18.3%	NA	Routinely	The evening of the operation day with at least 4 h of postsurgical observation
	OS	76	71	49 (17-83)	18.3%			The following day after surgery
Keulemans et al. [32]								
	DS	40	37	39 (20-62)	8.1%	Yes	Selectively	Discharged before 7 PM within the operation day
	OS	40	37	48 (19-65)	NA			At least one-night stay after surgery

DS: day surgery; OS: overnight stay; ^#^mean ± standard deviation; NA: not available; ^∗^*p* < 0.05.

## Supplementary Materials

Supplementary Materials Supplementary Figure S1: risk of bias summary and risk of bias graph. Supplementary S2: search strategy for “The Safety of Laparoscopic Cholecystectomy in Day Surgery Unit Comparing with That in the Inpatient Unit: A Systematic Review and Meta-Analysis.”
