# Supplementary material for: The Safety of Laparoscopic Cholecystectomy in the Day Surgery Unit Comparing with That in the Inpatient Unit: A Systematic Review and Meta-Analysis
Source: Biomed Res Int. 2020 Apr 28;2020:1924134. doi: 10.1155/2020/1924134 (PMC7206864; doi:10.1155/2020/1924134)
Supplement: Supplementary Materials — Supplementary Figure S1: risk of bias summary and risk of bias graph. Supplementary S2: search strategy for “The Safety of Laparoscopic Cholecystectomy in Day Surgery Unit Comparing with That in the Inpatient Unit: A Systematic Review and Meta-Analysis.” [file 1924134.f1.docx]

**Supplementary Material**

**The safety of laparoscopic cholecystectomy in day surgery unit comparing with that in the inpatient unit: a systematic review and meta-analysis**


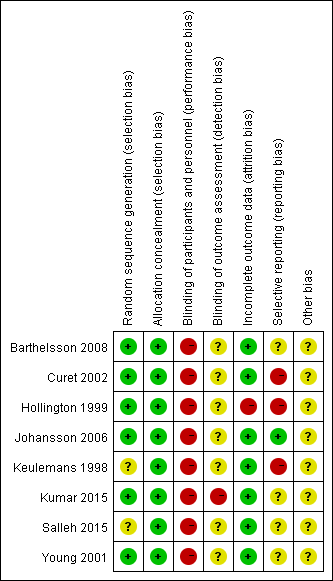

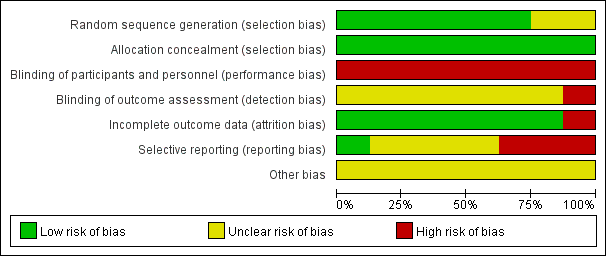


Figure s1. Risk of bias summary and risk of bias graph.

**Supplementary S2. Search strategy for The safety of laparoscopic cholecystectomy in day surgery unit comparing with that in the inpatient unit: a systematic review and meta-analysis.**

**Search strategy were developed following the five “PICOS” components.**

| PICOS-LC in day surgery unit vs. LC in the inpatient unit | |
| --- | --- |
| **P** | population: patients with benign diseases of gallbladder underwent LC |
| **I** | intervention: laparoscopic cholecystectomy (LC) |
| **C** | comparison: day surgery unit vs. inpatient unit |
| **O** | outcomes: Post-operative complications, post-operative nausea and vomiting (PONV) and visual analogue score (VAS), hospital stay, time to normal activity, patients’ satisfaction, readmission and cost. |
| S | study design: retrospective or prospective studies or randomized control trials |

**Embase.com 594**

('cholecystectomy'/exp OR (cholecystectom* OR (gallbladder* NEAR/3 (remov* OR resection*))):ab,ti) AND ('ambulatory surgery'/exp OR 'ambulatory care'/de OR outpatient/de OR 'outpatient department'/de OR 'outpatient care'/de OR (ambulatory OR outpatient* OR ((day) NEXT/1 (surger* OR case OR stay OR care OR hospital*)) OR same-day OR polyclinic* OR poly-clinic*):ab,ti) AND ('hospitalization'/exp OR 'hospital admission'/exp OR 'length of stay'/exp OR 'hospital patient'/exp OR 'hospital discharge'/de OR (hospitali* OR overnight* OR over-night* OR ((length OR hospital OR long OR short*) NEAR/3 stay) OR admission* OR admitted* OR inpatient* OR in-patient* OR routine OR discharge*):ab,ti) NOT ('case report'/de OR [Conference Abstract]/lim OR [Editorial]/lim OR [Erratum]/lim OR [Letter]/lim OR [Note]/lim OR 'case report':ti) AND [English]/lim NOT ([animals]/lim NOT [humans]/lim)

**Medline Ovid 498**

(exp Cholecystectomy/ OR (cholecystectom* OR (gallbladder* ADJ3 (remov* OR resection*))).ab,ti.) AND (Ambulatory Surgical Procedures/ OR Ambulatory Care/ OR Outpatients/ OR Ambulatory Care Facilities/ OR Outpatient Clinics, Hospital/ OR (ambulatory OR outpatient* OR ((day) ADJ (surger* OR case OR stay OR care OR hospital*)) OR same-day OR polyclinic* OR poly-clinic*).ab,ti.) AND (Hospitalization/ OR Length of Stay/ OR Inpatients/ OR (hospitali* OR overnight* OR over-night* OR ((length OR hospital OR long OR short*) ADJ3 stay) OR admission* OR admitted* OR inpatient* OR in-patient* OR routine OR discharge*).ab,ti.) NOT (case reports/ OR (news OR congres* OR abstract* OR book* OR chapter* OR dissertation abstract* OR letter* OR note OR editorial).pt. OR case report.ti.) AND english.la. NOT (exp animals/ NOT humans/)

**Web of science 442**

TS=(((cholecystectom* OR (gallbladder* NEAR/2 (remov* OR resection*)))) AND ((ambulatory OR outpatient* OR "day surger*" OR "day case" OR "day stay" OR "day care" OR "day hospital*" OR "same-day" OR polyclinic* OR poly-clinic*)) AND ((hospitali* OR overnight* OR over-night* OR ((length OR hospital OR long OR short*) NEAR/2 stay) OR admission* OR admitted* OR inpatient* OR in-patient* OR routine ORdischarge*))) AND DT=(article) AND LA=(english)

**Cochrane CENTRAL 125**

((cholecystectom* OR (gallbladder* NEAR/3 (remov* OR resection*))):ab,ti) AND ((ambulatory OR outpatient* OR ((day) NEXT/1 (surger* OR case OR stay OR care OR hospital*)) OR same next day OR polyclinic* OR poly next clinic*):ab,ti) AND ((hospitali* OR overnight* OR over next night* OR ((length OR hospital OR long OR short*) NEAR/3 stay) OR admission* OR admitted* OR inpatient* OR in next patient* OR routine OR discharge*):ab,ti)

**Google scholar**

cholecystectomy|"gallbladder removal|resection" ambulatory|outpatient|"day surgery|case|stay|care|hospital"|"same-day"|polyclinic hospitalization|overnight|admission|inpatient|discharge
